# Evolutionary dynamics of group formation

**DOI:** 10.1371/journal.pone.0187960

**Published:** 2017-11-14

**Authors:** Marco Alberto Javarone, Daniele Marinazzo

**Affiliations:** 1 School of Computer Science, University of Hertfordshire, Hatfield AL10 9AB, United Kingdom; 2 Dept. Mathematics and Computer Science, University of Cagliari, Cagliari 09123, Italy; 3 Department of Data Analysis, Faculty of Psychology and Educational Sciences, University of Ghent, Ghent, Belgium; 4 The Clinical Hospital of Chengdu Brain Science Institute, MOE Key Lab for Neuroinformation, University of Electronic Science and Technology of China, Chengdu, China; Public Library of Science, FRANCE

## Abstract

Group formation is a quite ubiquitous phenomenon across different animal species, whose individuals cluster together forming communities of diverse size. Previous investigations suggest that, in general, this phenomenon might have similar underlying reasons across the interested species, despite genetic and behavioral differences. For instance improving the individual safety (e.g. from predators), and increasing the probability to get food resources. Remarkably, the group size might strongly vary from species to species, e.g. shoals of fishes and herds of lions, and sometimes even within the same species, e.g. tribes and families in human societies. Here we build on previous theories stating that the dynamics of group formation may have evolutionary roots, and we explore this fascinating hypothesis from a purely theoretical perspective, with a model using the framework of Evolutionary Game Theory. In our model we hypothesize that homogeneity constitutes a fundamental ingredient in these dynamics. Accordingly, we study a population that tries to form homogeneous groups, i.e. composed of similar agents. The formation of a group can be interpreted as a strategy. Notably, agents can form a group (receiving a ‘group payoff’), or can act individually (receiving an ‘individual payoff’). The phase diagram of the modeled population shows a sharp transition between the ‘group phase’ and the ‘individual phase’, characterized by a critical ‘individual payoff’. Our results then support the hypothesis that the phenomenon of group formation has evolutionary roots.

## Introduction

The dynamics of group formation constitutes a topic of interest for a wide number of scientists, spanning from anthropologists to zoologists [1–7], and from social psychologists to economists [8–15]. In general, the formation of a group can be viewed as an emergent phenomenon [10, 16] where a number of individuals cluster together for performing one or more actions. Accordingly, the lifespan (as well as other characteristics) of a group can vary from case to case, and individuals can change group over time [17–19]. In an ecological system, being part of a group may allow to receive benefits [18], both as predator and as prey. For instance, the former can be advantaged during a hunt, e.g. surrounding a prey, while the latter can improve her/his safety staying inside a group [20]. In the case of humans, the previous example can be considered outdated. However, we should remind ourselves that millions of years ago, and maybe even in more recent times, humans have played both roles (i.e. predators and preys) in their ecosystem. Different studies suggest that the formation of social groups has evolutionary roots [1, 21–26], shared among animals belonging to different species. For instance, we can observe groups of fishes (generally named as shoals), of mammalians (named herds or families/tribes in the case of humans), and of birds (named flocks) [22].

What differs, from species to species, is the average size of a group [27–31], e.g. shoals are usually much bigger than herds, herds are bigger than families, and so on and so forth. In addition, even within the same species, groups can be of different size. The dynamics underlying their formation are of interest also beyond the domain of evolutionary biology, e.g. we can mention sport teams, business organizations [11], and scientific communities. Even if the motivations that lead to the formation of this kind of groups can be quite different from those that trigger the emergence of groups in nature, in both cases the individuals cluster together driven by a rational mindset, i.e. aimed to increase their wellness (or wealth). Therefore we believe that the framework of Evolutionary Game Theory (EGT hereinafter) [32–45] can be a suitable choice for studying this phenomenon, since it embodies both the rationality and the evolutionary aspects of group formation [46, 47]. In addition, we consider important to evaluate the role of similarity. In particular, the heterogeneity of a group can in principle constitute an advantage, or a disadvantage, depending on the context of reference (see for instance [48, 49]). Indeed, heterogeneity might refer to different aspects, as physical traits, genetic makeup, or skills. Previous studies (e.g. [50]) reported that social networks show a positive value of assortativity [51], i.e. individuals are more likely to connect with their own similar whereas, according to an entropic principle [50], other kinds of complex networks [52] are more likely to be disassortative. Thus, in the proposed model, we analyze an agent population that forms and breaks groups over time, according to the gain agents receive when act in group or individually. In particular, the gain comes from the difference between benefits and costs, in taking a particular action (i.e. staying in group, or acting individually). The gain achieved for being part of a group is defined ‘group payoff’, while that achieved by acting singularly is defined ‘individual payoff’. In addition, following the insights reported in [50], the ‘group payoff’ is maximized for homogeneous groups. Numerical simulations indicate that for each group size *G*, there is a critical ‘individual payoff’, that separates the ‘group phase’ from the ‘individual phase’ of a population, i.e. the clustering in groups and the retention of independent members. Moreover, results show that forming big groups is more difficult than forming small groups. To conclude, in our view, the achieved outcomes support the hypothesis of an evolutionary mechanism underlying the formation of groups in nature. Notably, we speculate that each animal species has its ‘individual payoff’, i.e. a kind of gain its individuals receive when they act as single members, and that this parameter might depend also on the considered environment. In addition, in the case of human beings, we suppose that the ‘individual payoff’ might be related also to socio-cultural conditions, leading to the formation of very small groups in the modern civilization, and to the formation of bigger groups (e.g. tribes) in more archaic systems (see [53–55]). Notably, two important differences between the modern civilization and the archaic ones are the living environment and the cultural structure (e.g. relations, laws, etc) of a society, both making a city more suitable than a forest for individual life styles.

## 1 Mathematical model

**Fig 1:**
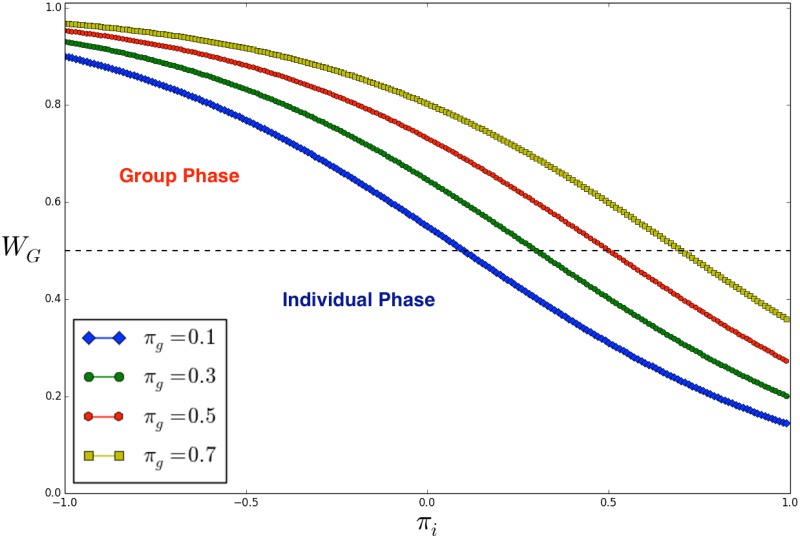
Probability distribution *W*_*G*_ in function of the individual payoff *π*_*i*_, as defined in Eq 1, considering four different values of *π*_*g*_ (see the legend). The black dotted line separates the ‘group phase’ from the ‘individual phase’, i.e. the values of *W*_*G*_ supporting the conservation of groups and those that lead to the emergence of individual behaviors.

In the proposed model, we consider a population with *N* agents that can form groups of size *G*. Each agent is represented by a spin vector *S*, of length *L*, e.g. for *L* = 6 the *i*-th agent can be represented as *S*_*i*_ = [+1, −1, −1, −1, +1, +1]. Here, each entry of the spin vector can be viewed as a feature, so the homogeneity of a group is measured considering the distance between the spin vectors of its members. We remark that, in this context, we use the concept of feature with its more general meaning, since it may vary from species to species. For instance, for many animals (including humans) a feature can be a physical trait, and in the case of humans it may represent also a hobby, or a specific skill, and so on (i.e. not only physical features). The dynamics of the proposed model is very simple. At each time step a number *G* of agents, not belonging to any group, is randomly selected. So, selected agents compute the potential payoff they could gain acting together (depending on the homogeneity of the potential group). In particular, the ‘group payoff’ *π*_*g*_ decreases when members have different spin vectors. Then, the value of *π*_*g*_ is compared to that of *π*_*i*_, i.e. the payoff that the same agents would gain acting individually. In doing so, the probability of forming a group (of size *G*), composed of the selected agents, depends on *π*_*i*_ and *π*_*g*_, and reads

$$\begin{array}{c} W_{G}={\left(1+\text{exp}\left[\frac{\pi_{i}-\pi_{g}}{K}\right]\right)}^{-1} \end{array}$$ (1)

where the constant *K* parametrizes the uncertainty in taking a decision (e.g. forming a group). By using *K* = 0.5, we implement a rational approach [35, 56]. After processing a new potential group, the model evaluates if a previous one, randomly selected among those formed at previous time steps, might be broken. The breaking process is performed according to the same equation adopted to generate a group (i.e. Eq 1). Fig 1 provides the illustration of Eq 1 for some fixed values of *π*_*g*_. Notably, we observe two different phases that can be reached by the population, i.e. ‘group phase’ and ‘individual phase’. The former implies that agents are able to form and to conserve groups of size *G*, while the latter implies that agents prefer to act as single individuals (i.e. breaking the groups after a while). It is now worth to clarify how to compute the group payoff in the proposed model. As mentioned before, the homogeneity of a group is computed according to the spin vectors of its members. Accordingly, the group payoff *π*_*g*_ is defined as the length of the normalized average sum of each spin vector (composing the considered group). In particular, since each entry can be positive (i.e. +1) or negative (i.e. −1), the absolute value of the average of a single spin is considered. The ‘group payoff’ for a group of size *G* and spin vectors of length *L* reads:

$$\begin{array}{c} \pi_{g}=\frac{1}{L}\frac{1}{G}\sum_{j=1}^{L}\left|\sum_{i=1}^{G}v_{ij}\right| \end{array}$$ (2)

with *v*_*i*_ elements of the spin vector of each agent. Additionally, it is worth noting that the range of *π*_*g*_ is [0, +1], while that of the ‘individual payoff’ *π*_*i*_ spans the interval [−1, +1]. The latter allows to represent scenarios where acting individually can be both very risky (i.e. *π*_*i*_ = −1), and very convenient (i.e. *π*_*i*_ = +1). At the same time, we assume that acting in group never leads to a negative payoff. Finally, we remind that during each simulation, the value of *π*_*i*_ remains constant. Summarizing, the proposed model can be described as follows:

1. At *t* = 0 generate a population providing each agent with a random spin vector;
2. While the number of time steps is smaller than *T*:
3. __ Randomly select *G* free agents (i.e. not belonging to other groups);
4. __ Compute the probability *W*_*G*_ (see Eq 1), i.e. selected agents form a new group;
5. __ Randomly select a group among those previously formed, and compute the probability to break it (by Eq 1);

Since we consider an asynchronous dynamics, i.e. only a subset of agents plays at a given time step, the value of *T* must be big enough in relation to the population size *N*.

**Fig 2:**
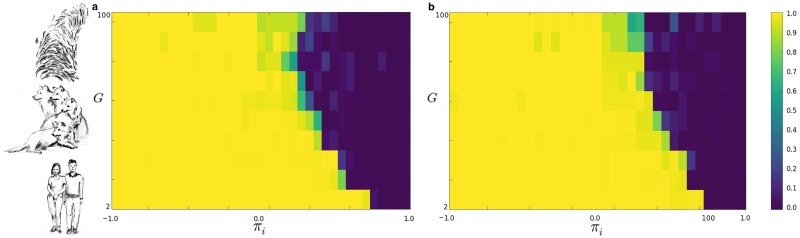
Phase diagram of the population, with groups of size *G* versus the ‘individual payoff’ *π*_*i*_, on varying the length of the spin vectors *L*. Yellow indicates the ‘group phase’, while Blue the ‘individual phase’. **a** *L* = 3 and **b** *L* = 10. The pictorial representation on the left aims to show groups of different size *G*, that we can observe in nature. Results have been averaged over different simulation runs.

**Fig 3:**
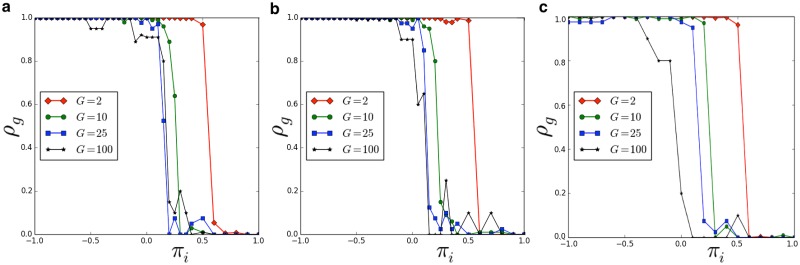
Density of groups *ρ*_*g*_ in function of the ‘individual payoff’ *π*_*i*_, on varying the length of the spin vectors *L*. **a**) *L* = 3. **b**) *L* = 10. **c**) *L* = 25. Results have been averaged over different simulation runs.

**Fig 4:**
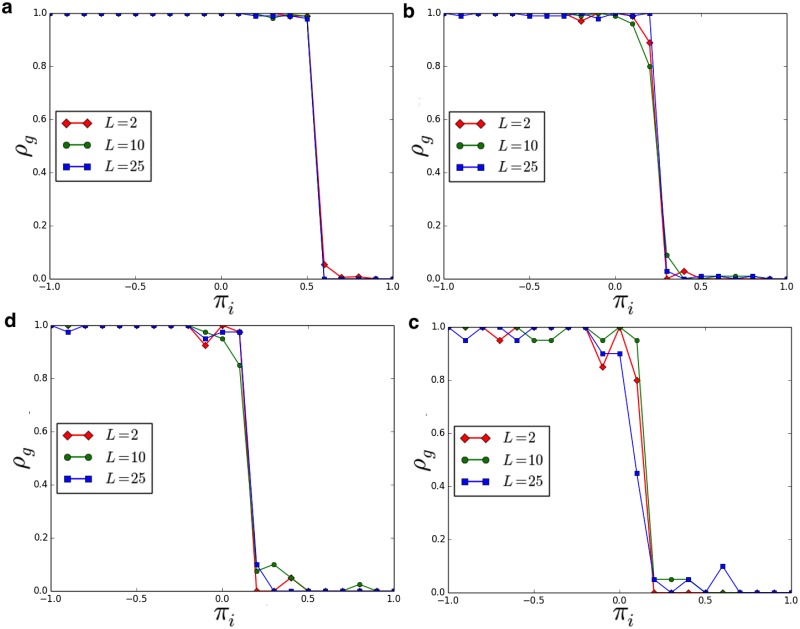
Density of groups *ρ*_*g*_ in function of the ‘individual payoff’ *π*_*i*_, for different spin vectors of length *L*, on varying the group size *G*. **a**) *G* = 2. **b**) *G* = 10. **c**) *G* = 25. **d**) *G* = 50. Results have been averaged over different simulation runs.

**Fig 5:**
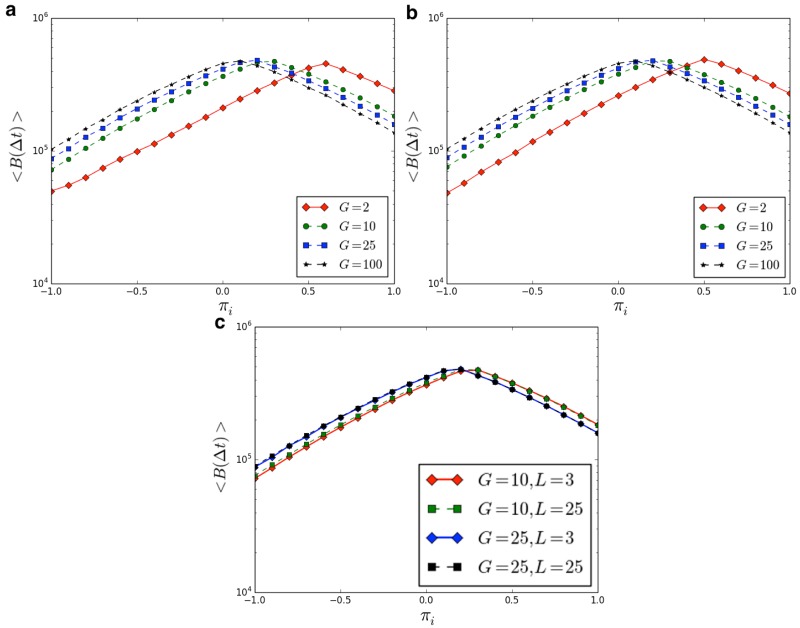
Average number of breaking groups < *B*(Δ*T*) > in the time interval Δ*T*. The legend indicates, for each line, the considered group size *G*. **a**) Results achieved with *L* = 3. **b**) Results achieved with *L* = 25. **c**) Comparison between results achieved with *L* = 3 and *L* = 25. Results have been averaged over different simulation runs.

Now we show results of numerical simulations (the source code for reproducing our results is available at [57].), performed in a population with *N* = 1000 agents, and considering different conditions related to the ‘group payoff’ and to the ‘individual payoff’ (i.e. *π*_*i*_ in the range [−1, +1] and *π*_*g*_ in the range [0, +1]). Moreover, we study the dynamics of the population for different lengths of the spin vector characterizing our agents. Due to the value of *N*, we analyzed the emergence of groups of the following size: [2, 4, 5, 10, 25, 50, 100]. Fig 2 illustrates the phase diagram of our population. Fig 3 indicates the density of the groups in function of the ‘individual payoff’, on varying the length of the spin vectors *L*. It is then possible to identify the critical thresholds $\hat{\pi_{i}}$, on varying the group size *G*. For instance, in the case *L* = 3, we observe $\hat{\pi_{i}}=0.55$ for *G* = 2, $\hat{\pi_{i}}=0.15$ for *G* = 10, and $\hat{\pi_{i}}=0.05$ for *G* = 25. Then, we evaluate if the length *L* (i.e. the length of the spin vectors) affects the outcomes of the model. As reported in Fig 4, it is interesting to observe that the density of groups (at equilibrium) is not affected by *L*. Eventually, we analyze the average number of breaking groups in the considered time interval Δ*T* —see Fig 5. In particular, we consider different group sizes *G*, and lengths *L*, on varying the individual payoff.

## 2 Discussion

In this work we studied the phenomenon of group formation using the framework of EGT. It is worth emphasizing that that one of the purposes of this investigation is to show that an abstract physical model can be used as a framework to study the interplay between evolution and group formation in nature. Notably, here we focus on the dynamics rather than on the correspondence between modeled agents and their real world counterpart, in the same way in which Ising spins are used to model large scale brain dynamics. In particular, we introduce a simple model where agents evaluate whether clustering together or acting individually, according to the payoff they receive taking one of these two actions. Under the assumption that the ‘group payoff’ (i.e. the gain received by forming a group) increases while increasing the homogeneity of a group, we study both the emergence and the breaking of groups. Even if further investigations would be required in order to evaluate the outcomes on varying the definition of the ‘group payoff’, as well as considering the group heterogeneity as dominant factor, we suppose that the achieved results can be considered general enough for envisioning some interesting speculations, related to the evolutionary aspects of group formation in nature. Notably, observing that groups form in species ranging from ants to birds, and from lions to human beings, we agree with the hypothesis that this process has evolutionary roots [6, 22]. In addition, we suggest that the ‘individual payoff’ is a relevant parameter representing the ensemble of genetic traits, skills, living environments, and even socio-cultural conditions one can observe in real systems. For instance, we hypothesize that being part of a group is more advantageous in a hostile environment than in a relaxed one, as suggested by some theories related to the formation of shoals of fishes. So, even considering the same species, some individuals act in very small groups, while others in bigger ones. For example, in the modern civilization [53, 55], small groups named families are, nowadays, composed of very few members, while tribes living in wilder environments are more copious. We emphasize that the proposed model suggests the existence of a critical threshold in the ‘individual payoff’, leading to a sharp transition in the phase diagram (see Fig 2), from a ‘group phase’ achieved for low values of *π*_*i*_ to an ‘individual phase’ achieved for high values of *π*_*i*_. Notably, for high values of the critical *π*_*i*_, group formation is scarcely observed. Here, ‘group phase’ and ‘individual phase’ correspond to the two states that the population can achieve at equilibrium, i.e. with agents forming groups or acting individually. In addition, it is worth clarifying that the critical threshold of *π*_*i*_, as shown in Fig 2, seems to depend on the size of the groups *G*. Notably, the smaller the value of *G*, the higher the critical *π*_*i*_. Eventually, results reported in Fig 5 confirm previous findings, and provide a further detail. In particular, analyzing the average number of breaking groups < *B*(Δ*t*) > in the considered time interval, we observe that small groups are more robust than big ones, and the maximum number of breaking groups is in correspondence with the critical threshold ${\hat{\pi }}_{i}$. Furthermore, for very high ‘individual payoffs’, big groups are more robust than small ones (i.e. the opposite of the case with low *π*_*i*_). Therefore, in agreement with previous investigations (e.g. [58, 59]), our results confirm the existence of a relation between the size of groups and the critical ‘individual payoff’. Before concluding, we deem of interest to provide some observations on the proposed model and on the achieved results, from the point of view of statistical physics. Our agents are characterized by a spin vector, and interact forming and breaking groups. Since the process of formation (and breaking) is faster than a thermalization process, the spin variables can be considered as *quenched*. In a real scenario, after a while, members belonging to the same group can learn from each other, thus a kind of ‘thermalization’ may be observed (i.e. agents belonging to the same group can become similar). On the other hand, allowing transitions from a disordered phase to an ordered one within one group, might prevent breaking processes. Further investigations might address this point, e.g. for studying a trade-off between converging time (i.e. thermalization) and number of breaking groups. Furthermore, we wish to focus on the role of the ‘individual-payoff’. In our view, the latter plays a role comparable to that of the temperature in the Ising model. Notably, for low temperatures the correlation length increases (i.e. spins converge to the same value), while for high temperatures the correlation length becomes very small. In our model, even if we cannot refer to the concept of correlation length due to the *quenched* state of spins, low values of *π*_*i*_ allow the emergence of groups (even big), while high values of *π*_*i*_ entail the agents tend to remain independent. Hence, even if for the reasons above reported, *π*_*i*_ is not properly a temperature and we cannot formally speak of a correlation length, we deem that the model speaks to an interpretation of the present also in the light of statistical physics. To conclude, we highlight that the proposed model represents an application of EGT besides its classical topics, as the emergence of cooperation, providing results that remarkably corroborate the hypothesis that the emergence of groups in animal species has evolutionary roots.
